# The impact of problematic mobile phone use and the number of close friends on depression and anxiety symptoms among college students

**DOI:** 10.3389/fpsyt.2023.1281847

**Published:** 2024-01-08

**Authors:** Wei Wang, Huiqiong Xu, Shuqin Li, Zhicheng Jiang, Yanjun Sun, Yuhui Wan

**Affiliations:** ^1^Department of Chinical Medicine, Suzhou Vocational Health College, Suzhou, Jiangsu, China; ^2^School of Public Health and Health Management, Anhui Medical College, Hefei, China; ^3^Department of Maternal, Child and Adolescent Health, School of Public Health, Anhui Medical University, Hefei, Anhui, China; ^4^Anhui Provincial Key Laboratory of Population Health and Aristogenics, Anhui Medical University, Hefei, Anhui, China; ^5^MOE Key Laboratory of Population Health Across Life Cycle, Hefei, Anhui, China; ^6^Public Health Department, Changfeng County Center for Disease Control and Prevention, Hefei, Anhui, China

**Keywords:** anxiety, depression, problematic mobile phone use, number of close friends, college students in China

## Abstract

**Background:**

Psychological problems often occur in college students, with the most common ones being depression and anxiety symptoms. Exploring the risk factors that influence depression and anxiety symptoms in college students is essential to promote their physical and mental health.

**Objective:**

This study aimed to investigate the independent and interaction effects of problematic mobile phone use (PMPU) and the number of close friends (NCFs) on depression and anxiety symptoms and the comorbidity of these symptoms among college students.

**Methods:**

A cross-sectional survey was conducted in Huainan, Anhui Province, and Suzhou, Jiangsu Province in China from October to December 2022. Data from 7,617 college students were collected. The Patient Health Questionnaire and Generalized Anxiety Disorder-7 were used to evaluate depression and anxiety symptoms. The PMPU data were collected by the Mobile Phone Addiction Type Scale. Multinomial logistic regression models were performed to examine the associations of PMPU and NCFs with depression and anxiety symptoms and their interaction effects.

**Results:**

PMPU and lack of close friends significantly increased the risk of depression and anxiety symptoms and the comorbidity of these symptoms in college students (*p* < 0.001). In addition, the effects of PMPU and lack of close friends on depression and anxiety symptoms in college students were interactive (*p* < 0.001). No significant sex differences were found.

**Conclusion:**

PMPU and lack of close friends are important risk factors for depression, anxiety, and the comorbidity of these symptoms in college students in China.

## 1 Introduction

College students are prone to depression and anxiety symptoms as they undergo many changes during their developmental stages, such as establishing new interpersonal relationships and adapting to new academic pressures (1, 2). A recent meta-analysis including 64 studies involving 100,187 individuals showed that the overall detection rates of depression and anxiety symptoms among university students were 33.6% (95% confidence interval CI., 29.3–37.8%) and 39.0% (95% CI, 34.6–43.4%) (3). Depression and anxiety symptoms were most commonly detected in low- and middle-income countries and among medical students (4, 5). College students with depression symptoms were associated with higher levels of suicidal behavior (6). High anxiety symptom scores among college students were associated with poor physical fitness (7).

Studies have shown that comorbidities often occur with psychiatric disorders; depression symptoms often coincide with anxiety symptoms (8). A British study evaluated anxiety and depression symptoms among college students and found a comorbidity rate of 27.8% (9). An Italian study evaluating anxiety and depression symptoms among medical students at two universities found that 47% reported comorbid symptoms of anxiety and depression (10). In addition, a Chinese study evaluating anxiety and depression symptoms among college students showed that 18.3% had comorbid symptoms of anxiety and depression (11). Compared with students with either depression or anxiety symptoms, college students with comorbid anxiety and depression usually experience more stressful life events, worse emotional regulation ability, more severe physical and mental symptoms, more serious impairment of social functioning, and worse prognosis, making the comorbidities of depression and anxiety a key public health issue (12). As a result, there is increasing attention being paid to the factors that contribute to depression and anxiety symptoms, the comorbidity of these symptoms, as well as the interactions among these factors (13).

The popularity and convenience of smartphone use can lead to psychological problems. Problematic mobile phone use (PMPU) is an addictive behaviour caused by the excessive use of mobile phones, which impairs the psychological and social functions of users, enables mobile phone dependence and negatively affects daily lives (14). Domestic and foreign studies have shown that excessive or frequent use of mobile phones can cause wrist and neck pain (15, 16), blurred vision (17, 18), poor academic performance (19), and poor sleep quality (20, 21). Recently, many studies have focused on the effects of mobile phone overuse on mental health. Some researchers found that mobile phone overuse positively correlates with depression and anxiety scores among college students, indicating that college students with more severe mobile phone overuse are more likely to experience depression and anxiety symptoms (22–25). These findings suggest that PMPU is an important risk factor for depression and anxiety. Given that depression and anxiety symptoms often occur simultaneously, this study focused on the effects of PMPU on the comorbidity of depression and anxiety.

Friendship is a valuable source of social support throughout life and provides psychological support to people facing stressful events. Previous studies have found that a lack of close friends is significantly associated with depression and anxiety symptoms among college students (26). Evidence suggests that the quantity and quality of social relationships affect various health outcomes, including mental health (27). In addition, the World Health Organization suggests that developing interpersonal skills among adolescents can help reduce mental health problems such as depression and anxiety. Depression and anxiety can lead to broader adverse effects on adolescents physical and mental health as well as adverse health and social consequences (28). This study focused on the association between the number of close friends (NCFs) and depression and anxiety. Further, it explored the role of the number of close friends on the comorbidity of depression and anxiety.

Previous research has shown that PMPU is associated with psychological problems in college students and that good peer relationships are an important protective factor from mobile phone addiction and mental health in adolescents. However, the association between PMPU and NCF interactions on depression and anxiety symptoms in college students has not been determined. Therefore, this study investigated the prevalence of depression and anxiety symptoms and their comorbidities. Second, the independent and interaction effects of PMPU and NCFs on depression and anxiety symptoms in college students were analysed.

## 2 Materials and methods

### 2.1 Aim and design

This study investigated the moderating effect of NCFs on the relationship between PMPU, depression, and anxiety symptoms among college students. In October 2022, we conducted a cross-sectional survey at two colleges in Huainan, Anhui Province, and Suzhou, Jiangsu Province, China.

### 2.2 Participants

We conducted an electronic questionnaire for all first-year students. After screening, 249 invalid questionnaires were removed, because 1.2% (93) of the students or their parents/guardians were unwilling to participate in the study/investigation,0.6% (29) students were absent on the day of the survey, and 1.3% (105) has incomplete ques. tionnaires with a high level of missing data (>15%) or apparent logic errors or inconsistent answers. Ultimately, 7,617 valid samples were obtained, at an efciency rate of 96.8%. According to the previous survey results of the author’s research team, the prevalence of depression, anxiety, and co-morbidity of depression and anxiety symptoms were 20.0, 20.3, and 10.1%, respectively (13), and the calculation of study power inferred that 7,617 subjects could meet the needs of the design sample size of this study. A total of 2,312 (30.4%) were boys, and 5,305 (69.6%) were girls. The mean age was 18.9 ± 0.84 years. This study was approved by the Suzhou Health Vocational and Technical College (batch number: SW-YXLL202202). Informed consent was obtained from participants, all participants signed informed consent forms.

### 2.3 Measures

Data on participant demographics, problematic mobile phone use, depression, and anxiety symptoms were collected using electronic questionnaires.

#### 2.3.1 Demographic information

Information was collected on sex, place of residence, only child status, family economic situation, parent education level, and NCFs. The type of family residence was classified as either urban or rural. Family economic status was classified as low, medium, or high. The educational level of parents was divided into two categories: junior high school and below or high school and above.

#### 2.3.2 Problematic mobile phone use

PMPU was measured using the Mobile Phone Addiction Type Scale, developed by Xiong et al. (30). This scale has been widely used to evaluate the PMPU of college students and has shown good reliability and validity in China (31). The scale includes 16 items corresponding to four dimensions: withdrawal symptoms, significance, social comfort, and mood changes. All items are evaluated on a 5-point scale, ranging from 1 (never) to 5 (always). The total score is the sum of the scores of the four dimensions, ranging from 16 to 80 points. The higher the total score, the higher the PMPU level. In this study, Cronbach’s α coefficient for this scale was 0.94.

#### 2.3.3 The number of close friends

NCFs were measured using the question, ‘How many close friends do you have?’ which is a valid measure that has been used in a lot of studies (32, 33). The NCFs were divided into three categories: 0, 1–2 and ≥ 3.

#### 2.3.4 Depression and anxiety symptoms

Depression symptoms were assessed using the 9-item Patient Health Questionnaire-9 (PHQ-9). This scale is derived from the Diagnostic and Statistical Manual of Mental Disorders (34). The PHQ-9 scale score is the sum of the scores for each item from 1 to 9, and the total score range of the PHQ-9 is 0 to 27. The total score of PHQ-9 can be used to assess the depression symptoms: < 5 is no depression symptoms, and ≥ 5 is depression symptoms. The PHQ-9 has shown good reliability (Cronbach’s α = 0.87) and effectiveness in previous studies (35) and has been widely used with Chinese college students (36). Cronbach’s α coefficient in this study was 0.90.

Anxiety symptoms were assessed using the 7-item Generalized Anxiety Disorder-7 (GAD-7) developed by Spitzer et al. (37). The total GAD-7 score can be used to assess the presence or absence of anxiety symptoms: < 5 is no anxiety symptoms, and ≥ 5 is anxiety symptoms. The GAD-7 has shown good reliability (Cronbach’s α of 0.88) and effectiveness in previous studies (38) and has been widely used with Chinese college students (39). Cronbach’s α coefficient of this study is 0.95. If both depressive and anxiety symptoms are present, it is determined to be comorbid with depression and anxiety; if you have only anxiety or depression symptoms, you are anxious or depressed.

### 2.4 Statistical analysis

All analyses were conducted using the SPSS software (version 23.0; SPSS Inc., Chicago, IL, USA). First, the chi-square test was used to compare sex differences among demographic variables. Second, multivariate logistic regression models were performed to examine the associations and evaluate the interactions between PMPU, NCFs, depression symptoms, anxiety symptoms, and comorbidity of depression and anxiety symptoms. Adjustments were made for confounding factors, such as sex, place of residence, only child status, family economic situation, and parent education level. The odds ratios (OR) and 95% CI for these factors were calculated to determine their associations. Third, the adjusted model effects were tested for different sex subgroups. Finally, sex differences in the associations were examined using the ratio of the two odds ratios (40). A *p* value of <0.05 indicated statistical significance.

## 3 Results

### 3.1 Characteristics of the participants

Among the 7,617 participants, 69.6% (n = 5,305) were female students. The prevalence of depression symptoms, anxiety symptoms, and comorbidity of depression and anxiety symptoms was 49.9, 34.9, and 32.3%, respectively (Table 1).

**Table 1:** Characteristic of participants by gender, data shown as n% / M(SD).

Variables	Total N = 7,617	Boy n_1_ = 2,312	Girl n_2_ = 5,305	χ*^2^*	*P*
Residence				9.77	0.002
Rural	4,100(53.8)	1,307(56.5)	2,793(52.6)		
Urban	3,517(46.2)	1,005(43.5)	2,512(47.4)		
Only child				34.21	<0.001
Yes	2074(27.2)	734(35.4)	1,340(64.6)		
No	5,543(72.8)	1,578(28.5)	3,965(71.5)		
Family economic status				95.21	<0.001
Low	1978(26.0)	770(38.9)	1,208(61.1)		
Medium	5,252(69.0)	1,423(27.1)	3,829(72.9)		
High	387(5.1)	119(30.7)	268(69.3)		
Father’s education level				19.18	<0.001
Junior middle school and below	4,441(58.3)	1,334(30.3)	3,107(70.0)		
Senior middle school and above	2,497(32.8)	723(29.0)	1774(71.0)		
Unclear	679(8.9)	255(37.6)	424(62.4)		
Mother’s education level				64.41	<0.001
Junior middle school and below	4,750(62.4)	1,395(29.4)	3,355(70.6)		
Senior middle school and above	2014(26.4)	558(27.7)	1,456(72.3)		
Unclear	853(11.2)	359(42.1)	494(57.9)		
NCFs				41.55	<0.001
0	253(3.3)	114(45.1)	139(54.9)		
1 ~ 2	2,912(38.2)	795(27.3)	2,117(72.7)		
≥3	4,452(58.4)	1,403(31.5)	3,049(68.5)		
Problematic mobile phone use				24.92	<0.001
No	5,596(73.5)	1787(31.9)	3,809(68.1)		
Yes	2021(26.5)	525(26.0)	1,496(74.0)		
Depression symptoms				46.70	<0.001
No	3,818(50.1)	1,296(33.9)	2,522(66.1)		
Yes	3,799(49.9)	1,016(26.7)	2,783(73.3)		
Anxiety symptoms				30.59	<0.001
No	4,959(65.1)	1,611(32.5)	3,348(67.5)		
Yes	2,658(34.9)	701(26.4)	1957(73.6)		
Depression and anxiety symptoms				29.13	<0.001
No	5,155(67.7)	1,666(32.3)	3,489(67.7)		
Yes	2,462(32.3)	646(26.2)	1816(73.8)		

### 3.2 Multivariate logistic regression analyses

Results from multivariate logistic regression analysis indicated that both PMPU (OR = 6.14, 95% CI: 5.44–6.93; OR = 5.27, 95% CI: 4.73–5.88; OR = 9.03, 95% CI: 7.90–10.33) and NCFs (OR_0_ = 1.97, 95% CI: 1.52–2.56, OR_1-2_ = 1.52, 95% CI: 1.38–1.67; OR_0_ = 1.96, 95% CI: 1.52–2.53, OR_1-2_ = 1.56, 95% CI: 1.41–1.72; OR_0_ = 2.28, 95% CI: 1.72–3.03, OR_1-2_ = 1.69, 95% CI: 1.52–1.88) remained independently associated with depression symptoms, anxiety symptoms, comorbidity of depression and anxiety symptoms (*p* < 0.001 for each, Supplementary Table S1). In addition, they had a multiple interaction impact on depression symptoms, anxiety symptoms, and comorbidity of depression and anxiety symptoms. PMPU_yes_ + NCF_0_ (OR = 10.16, 95% CI: 5.08–20.323, OR = 6.52, 95% CI: 4.00–10.64, OR = 6.20, 95% CI: 3.87–9.93) and PMPU_yes_ + NCF_1-2_ (OR = 6.38, 95% CI: 5.281–7.717, OR = 4.98, 95% CI: 4.27–5.81, OR = 5.11, 95% CI: 4.39–5.95) was associated with depression symptoms, anxiety symptoms, comorbidity of depression and anxiety symptoms (*p* < 0.001 for each, Supplementary Table S1). After adjusting for sex, residence, only child status, family economic status, and parent education level, these positive associations remained significant (Table 2).

**Table 2:** The effects of PMPU and NCFs on depression and anxiety symptoms, adjusted model.

Variables	Depression symptoms		Anxiety symptoms		Depression and anxiety symptoms	
	*OR*(95*CI*%)*	*P*	*OR*(95*CI*%)*	*P*	*OR*(95*CI*%)*	*P*
PMPU						
no	1.00		1.00		1.00	
yes	6.09(5.39–6.88)	<0.001	5.21(4.67–5.81)	<0.001	5.46(4.89–6.10)	<0.001
NCF						
0	1.90(1.45–2.48)	<0.001	1.89(1.46–2.46)	<0.001	1.99(1.53–2.58)	<0.001
1–2	1.46(1.33–1.61)	<0.001	1.51(1.37–1.67)	<0.001	1.50(1.36–1.66)	<0.001
≥3	1.00		1.00		1.00	
PMPU×NCF						
no× ≥ 3	1.00		1.00		1.00	
yes×0	9.45(4.71–18.95)	<0.001	6.04(3.69–9.90)	<0.001	5.72(3.56–9.20)	<0.001
yes×1–2	6.20(5.12–7.50)	<0.001	4.86(4.16–5.67)	<0.001	4.98(4.28–5.81)	<0.001

*Adjusting for sex, residence, only child, family economic status, parents’ education level; PMPU, problematic mobile phone use; NCFs, the number of close friends.

### 3.3 Differences between sex subgroups

**Figure 1:**
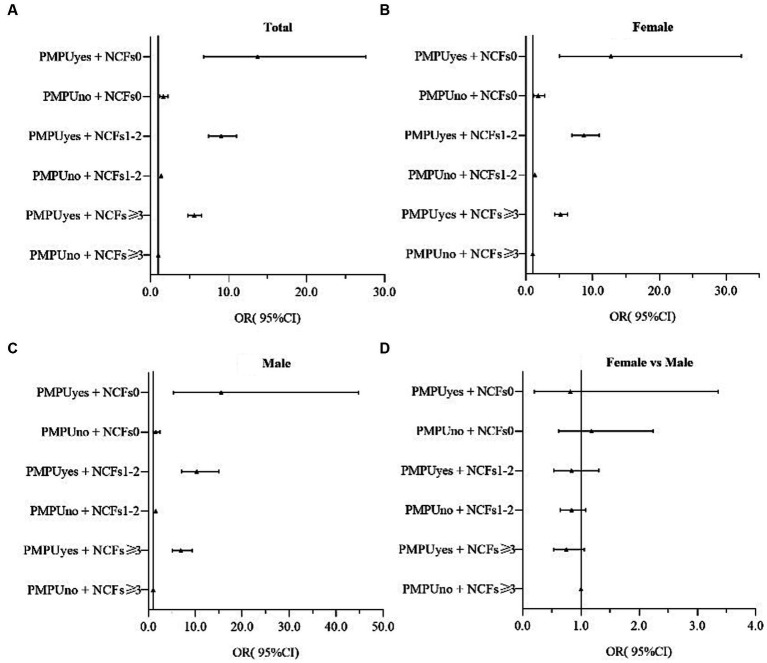
OR(95%CI) associated of PMPU and NCF on depression symptoms in female and male, and the gender ratio. PMPU = problematic mobile phone use; NCFs = the number of close friends. Adjusted for sex, residence, only child, family economic status, parents’ education level. **(A)** All participants, **(B)** Male participants, **(C)** Female participants, **(D)** Male participants compared to female participants.

**Figure 2:**
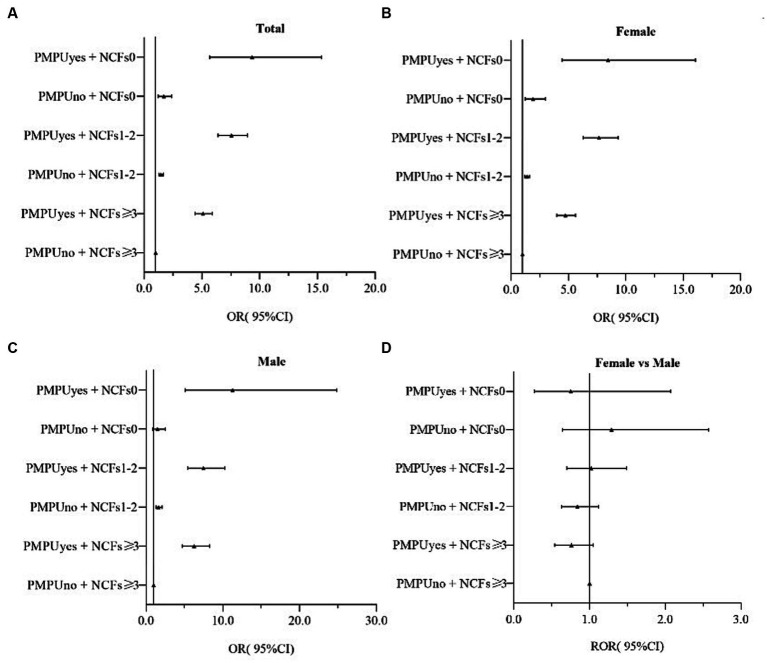
OR(95%CI) associated of PMPU and NCFs on anxiety symptoms in female and male, and the gender ratio. PMPU = problematic mobile phone use; NCFs = the number of close friends. Adjusted for sex, residence, only child, family economic status, parents’ education level. **(A)** All participants, **(B)** Male participants, **(C)** Female participants, **(D)** Male participants compared to female participants.

**Figure 3:**
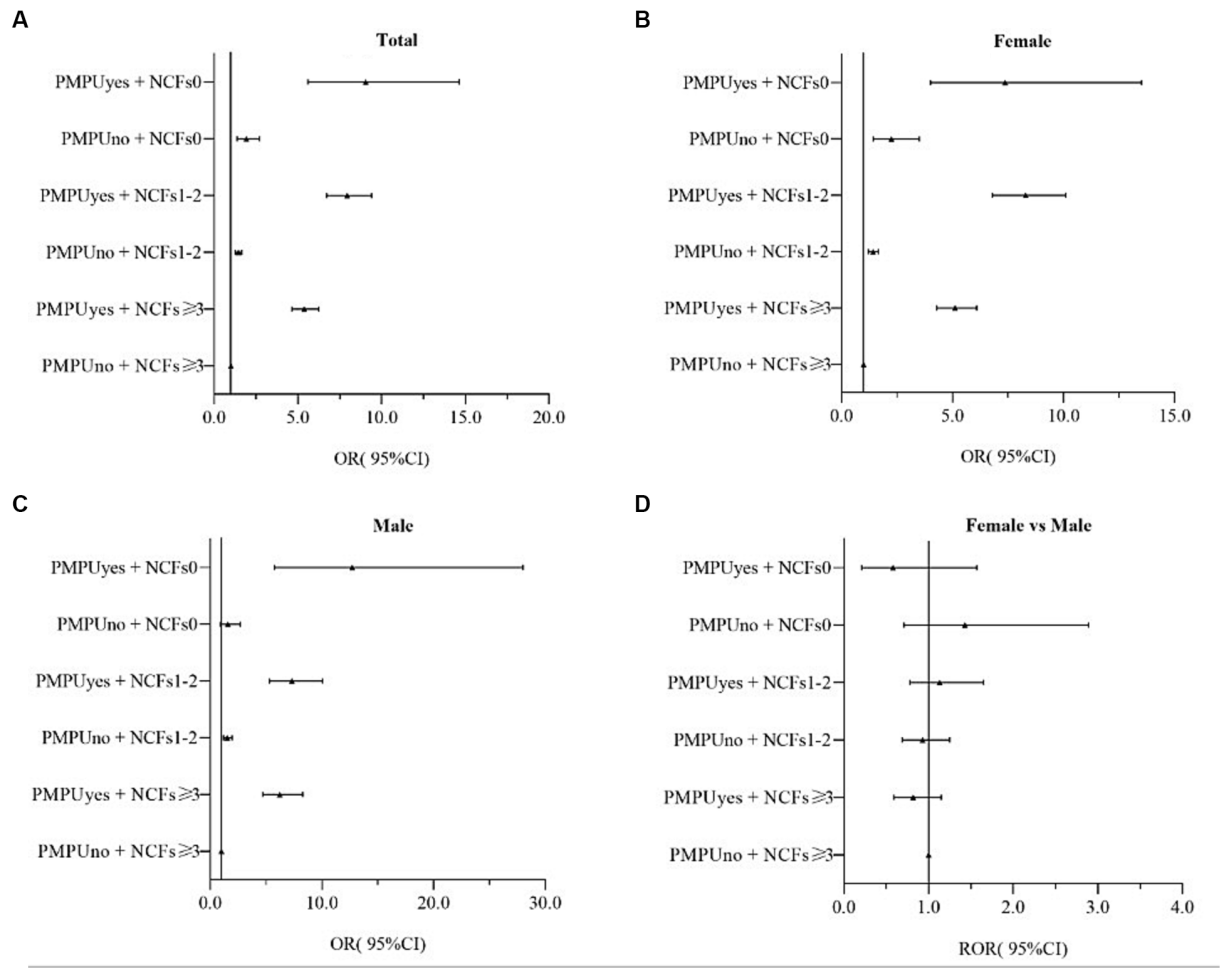
OR(95%CI)associated of PMPU and NCFs on depression and anxiety symptoms in female and male, and the gender ratio. PMPU = problematic mobile phone use; NCFs = the number of close friends. Adjusted for sex, residence, only child, family economic status, parents’ education level. **(A)** All participants, **(B)** Male participants, **(C)** Female participants, **(D)** Male participants compared to female participants.

In both boys and girls, after adjusting for sex, residence, only child status, family economic status, and parent education level, PMPU and NCFs remained independently associated with depression symptoms (Figure 1), anxiety symptoms (Figure 2), comorbidity of depression and anxiety symptoms (Figure 3). In addition, they had multiple interacting effects on depressive symptoms, anxiety symptoms, and comorbidities of depression and anxiety symptoms. However, no statistically significant differences were found between the sexes. The specific values were detailed in supplementary material (Supplementary Tables S2–S4).

## 4 Discussion

The results of this study showed that the detection rate of depression symptoms in college students was 49.9%, which was higher than previous results on depression symptoms among Chinese college students (20%) (41). In addition, relevant studies have shown that the detection rate of depression symptoms among college students was in Association of Southeast Asian Nations (29.4%) (42), Tanzania (21.3%) (43), Canada (8%) (44), and Australia (21.8%) (45). The detection rate of anxiety symptoms in this study was 34.9%, which was higher than the results of a study on anxiety symptoms in Chinese college students (30.8%) (46) and similar to the results of a meta-analysis of 69 studies involving 40,348 college students (33.8%) (47). Other studies showed that the detection rates of anxiety symptoms among college students were in Israel (29.4%) (48), Spain (23.6%) (49), and Japan (30.5%) (50). The prevalence of depression and anxiety comorbidities in the participants in this study was 32.3%, which was higher than the previous detection rate of depression and anxiety comorbidities among Chinese college students (20.9%) (51), Ethiopian college students (20.0%) (29), and American college students (10.0%) (52). These inconsistent results may be related to the different survey periods, respondents, and questionnaires. The study was completed during the COVID-19 epidemic, and the participants were college students. The use of PHQ-9 and GAD-7 to evaluate depression and anxiety symptoms may be the main reason for the high detection rate of depression and anxiety symptoms in this study (53–58). In addition, the incidence of depression, anxiety, and comorbidities was higher in girls than in boys in this study, possibly due to biological structure and social roles (3).

This study showed that PMPU is an independent risk factor for depression and anxiety symptoms in college students. Previous studies have shown that negative emotions such as depression symptoms are predictors of mobile phone addictive behaviour (59, 60). According to behavioural cognitive theory, an individual not only reacts to emotions but also responds to their emotions (61). Increasing evidence shows that PMPU is an important risk factor for depression and anxiety symptoms in college students, endangering their physical and mental health (62, 63). A meta-analysis of 40 studies involving 33,650 university students showed that PMPU was positively associated with anxiety and depression (64). Studies have shown that PMPU can cause symptoms of depression and anxiety in college students by affecting their sleep quality (65). In addition, lack of close friends is another important risk factor for depression and anxiety symptoms among college students. According to the interpersonal relationship theory, interpersonal problems increase social anxiety, reducing interpersonal security and increasing the risk of depression (66). Evidence has shown that a lack of close friends predisposes students to poor emotional states, leading to depression, anxiety, or both (67). Increased intimate peer contact has been associated with fewer anxiety symptoms in men and women and fewer depressive symptoms in women (68).

Earlier research has confirmed that technology addiction (e.g., mobile phone addiction) is often accompanied by relationship problems (69). The displacement theory states that if an individual has a compulsive need to use a mobile phone, it may reduce face-to-face social circles and communication, leading to a lack of close friends and, ultimately, more interpersonal problems (70). Studies have pointed out that unlike chemical addictions (e.g., drugs), PMPU is a technological addiction that makes it difficult to directly affect the psychiatric problems of an individual without other moderating variables (71). The results showed that PMPU interacted with a lack of close friends to jointly influence depression and anxiety symptoms in college students. PMPU and interpersonal relationship problems, such as lack of close friends, are two-way causal and produce a vicious cycle, affecting depression and anxiety symptoms (71). In addition, studies have shown that close friends are an important factor in influencing and maintaining physical activity during college (72), and moderate-to-high-intensity physical activity is associated with a reduced risk of depression symptoms in students (73). It was indirectly confirmed that PMPU interacts with a lack of close friends, affecting the mental health of college students. This study did not find a sex difference in the effect of PMPU and lack of close friends on depression and anxiety symptoms, prompting us to focus on the effects of PMPU and NCFs on the physical and mental health of college students.

## 5 Strengths and limitations

Although this study elucidated the effect of the interaction between PMPU and NCFs on depression and anxiety symptoms in college students, it also focused on independent and interactive effects on depression and anxiety comorbidities. However, this study has several limitations. First, it was conducted during the COVID-19 pandemic. In the context of COVID-19, Chinese college students needed to complete learning tasks online, which inevitably increased the risk of developing a dependence on mobile phones. However, owing to data limitations, we did not measure the effect of using mobile phones for learning in online courses. Future research should attempt to avoid the impact of online courses on mobile phone usage under special environmental conditions. Second, this is a cross-sectional study with evidence of a two-way relationship between PMPU and depression and anxiety symptoms. Therefore, a causal relationship cannot be inferred, and longitudinal studies should be considered and mediation effect analysis should be attempted to test the size of the mediating effect of PMPU and NCFs on individuals. Third, our sample was from a university campus, the distribution of male and female students was uneven, and the selection bias of the sample may limit the interpretation of the results. Future studies should be extended to the community and include an adolescent sample to provide a theoretical basis for promoting the physical and mental health of more Chinese adolescents. Finally, this study relied on retrospective self-reporting from questionnaires, and students may have recall bias when completing the questionnaire. Future research should develop detailed data collection and strict quality control methods.

## 6 Conclusion

PMPU and lack of close friends are important risk factors for depression and anxiety symptoms in college students. In addition, PMPU has a synergistic effect on depression and anxiety symptoms in the absence of close friends. Therefore, college students with PMPU who lack close friends are more likely to have symptoms of depression and anxiety. Paying attention to PMPU situations and interpersonal relationships among college students can help reduce depression and anxiety symptoms.

## Data availability statement

The raw data supporting the conclusions of this article will be made available by the authors, without undue reservation.

## Ethics statement

The studies involving humans were approved by Suzhou Vocational Health College Ethics Committee. The studies were conducted in accordance with the local legislation and institutional requirements. The participants provided their written informed consent to participate in this study.

## Author contributions

WW: Data curation, Funding acquisition, Writing – original draft. HX: Writing – original draft, Writing – review & editing. SL: Data curation, Investigation, Resources, Writing – review & editing. ZJ: Supervision, Validation, Writing – review & editing. YS: Validation, Writing – review & editing. YW: Data curation, Investigation, Resources, Writing – review & editing.

## References

[1] Liu Y Zhang N Bao G Huang Y Ji B Wu Y. Predictors of depressive symptoms in college students: a systematic review and meta-analysis of cohort studies. J Affect Disord. (2019) 244:196–208. doi: 10.1016/j.jad.2018.10.084, PMID: 30352363

[2] Naja WJ Kansoun AH Haddad RS. Prevalence of depression in medical students at the Lebanese University and exploring its correlation with Facebook relevance: a questionnaire study. JMIR Res Protoc. (2016) 5:e96. doi: 10.2196/resprot.4551, PMID: 27246394 PMC4908302

[3] Li W Zhao Z Chen D Peng Y Lu Z. Prevalence and associated factors of depression and anxiety symptoms among college students: a systematic review and meta-analysis. J Child Psychol Psychiatry. (2022) 63:1222–30. doi: 10.1111/jcpp.13606, PMID: 35297041

[4] Akhtar P Ma L Waqas A Naveed S Li Y Rahman A. Prevalence of depression among university students in low and middle income countries (LMICs): a systematic review and meta-analysis. J Affect Disord. (2020) 274:911–9. doi: 10.1016/j.jad.2020.03.183, PMID: 32664032

[5] Azad N Shahid A Abbas N Shaheen A Munir N. Anxiety and depression in medical students of a private medical college. J Ayub Med Coll Abbottabad. (2017) 29:123–7. PMID: 28712190

[6] Casey SM Varela A Marriott JP Coleman CM Harlow BL. The influence of diagnosed mental health conditions and symptoms of depression and/or anxiety on suicide ideation, plan, and attempt among college students: findings from the healthy minds study, 2018-2019. J Affect Disord. (2022) 298:464–71. doi: 10.1016/j.jad.2021.11.006 34774646

[7] Yin J Kong L Cui Y. Association analyses of physical fitness parameters and anxiety symptoms in Chinese college students. Int J Environ Res Public Health. (2022) 20:623. doi: 10.3390/ijerph20010623, PMID: 36612943 PMC9820032

[8] Lamers F van Oppen P Comijs HC Smit JH Spinhoven P van Balkom AJ. Comorbidity patterns of anxiety and depressive disorders in a large cohort study: the Netherlands study of depression and anxiety (NESDA). J Clin Psychiatry. (2011) 72:341–8. doi: 10.4088/JCP.10m06176blu 21294994

[9] Jenkins PE Ducker I Gooding R James M Rutter-Eley E. Anxiety anddepression in a sample of UK college students: a study ofprevalence, comorbidity, and quality of lifell. J Am Coll Heal. (2021) 69:813–9. doi: 10.1080/07448481.2019.1709474, PMID: 31995452

[10] Bertani DE Mattei G Ferrari S Pingani L Galeazzi GM. Anxiety, depression and personality traits in Italian medical students. Riv Psichiatr. (2020) 55:342–8. doi: 10.1708/3503.34892, PMID: 33349727

[11] Cheng S Jia C Wang Y. Only children were associated with anxiety and depressive symptoms among college students in China. Int J Environ Res Public Health. (2020) 17:4035. doi: 10.3390/ijerph17114035, PMID: 32517044 PMC7313008

[12] Choi KW Kim YK Jeon HJ. Comorbid anxiety and depression: clinical and conceptual consideration and Transdiagnostic treatment. Adv Exp Med Biol. (2020) 1191:219–35. doi: 10.1007/978-981-32-9705-0_14, PMID: 32002932

[13] Zhang Y Li S Xu H Jin Z Li R Zhang Y. Gender-based differences in interaction effects between childhood maltreatment and problematic mobile phone use on college students' depression and anxiety symptoms. BMC Psychiatry. (2023) 23:286. doi: 10.1186/s12888-023-04777-x, PMID: 37098541 PMC10127168

[14] Yen CF Tang TC Yen JY Lin HC Huang CF Liu SC. Symptoms of problematic cellular phone use, functional impairment and its association with depression among adolescents in southern Taiwan. J Adolesc. (2009) 32:863–73. doi: 10.1016/j.adolescence.2008.10.006, PMID: 19027941

[15] Baabdullah A Bokhary D Kabli Y Saggaf O Daiwali M Hamdi A. The association between smartphone addiction and thumb/wrist pain: a cross-sectional study. Medicine (Baltimore). (2020) 99:e19124. doi: 10.1097/MD.0000000000019124, PMID: 32150053 PMC7478614

[16] Tsantili AR Chrysikos D Troupis T. Text neck syndrome: disentangling a new epidemic. Acta Med Acad. (2022) 51:123–7. doi: 10.5644/ama2006-124.380, PMID: 36318004 PMC9982850

[17] Almudhaiyan TM Aldebasi T Alakel R Marghlani L Aljebreen A Moazin OM. The prevalence and knowledge of digital eye strain among the undergraduates in Riyadh, Saudi Arabia. Cureus. (2023) 15:e37081. doi: 10.7759/cureus.37081, PMID: 37153239 PMC10156438

[18] Chu GCH Chan LYL Do CW Tse ACY Cheung T Szeto GPY. Association between time spent on smartphones and digital eye strain: a 1-year prospective observational study among Hong Kong children and adolescents. Environ Sci Pollut Res Int. (2023) 30:58428–35. doi: 10.1007/s11356-023-26258-0, PMID: 36991204 PMC10057686

[19] Liu X Luo Y Liu ZZ Yang Y Liu J Jia CX. Prolonged mobile phone use is associated with poor academic performance in adolescents. Cyberpsychol Behav Soc Netw. (2020) 23:303–11. doi: 10.1089/cyber.2019.0591, PMID: 32191529

[20] Moreno MA. Media use and sleep. JAMA Pediatr. (2016) 170:1236. doi: 10.1001/jamapediatrics.2015.2575 27918791

[21] Rathakrishnan B Bikar Singh SS Kamaluddin MR Yahaya A Mohd Nasir MA Ibrahim F. Smartphone addiction and sleep quality on academic performance of university students: an exploratory research. Int J Environ Res Public Health. (2021) 18:8291. doi: 10.3390/ijerph18168291, PMID: 34444042 PMC8394437

[22] Višnjić A Veličković V Sokolović D Stanković M Mijatović K Stojanović M. Relationship between the manner of mobile phone use and depression, anxiety, and stress in university students. Int J Environ Res Public Health. (2018) 15:697. doi: 10.3390/ijerph15040697, PMID: 29642471 PMC5923739

[23] El-Sayed Desouky D Abu-Zaid H. Mobile phone use pattern and addiction in relation to depression and anxiety. East Mediterr Health J. (2020) 26:692–9. doi: 10.26719/emhj.20.043, PMID: 32621504

[24] Yang J Fu X Liao X Li Y. Association of problematic smartphone use with poor sleep quality, depression, and anxiety: a systematic review and meta-analysis. Psychiatry Res. (2020) 284:112686. doi: 10.1016/j.psychres.2019.112686, PMID: 31757638

[25] Elhai JD Yang H McKay D Asmundson GJG. COVID-19 anxiety symptoms associated with problematic smartphone use severity in Chinese adults. J Affect Disord. (2020) 274:576–82. doi: 10.1016/j.jad.2020.05.080, PMID: 32663990 PMC7251360

[26] Badr HE Naser J Al-Zaabi A Al-Saeedi A Al-Munefi K Al-Houli S. Childhood maltreatment: a predictor of mental health problems among adolescents and young adults. Child Abuse Negl. (2018) 80:161–71. doi: 10.1016/j.chiabu.2018.03.011, PMID: 29609135

[27] Umberson D Montez JK. Social relationships and health: a flashpoint for health policy. J Health Soc Behav. (2010) 51:S54–66. doi: 10.1177/0022146510383501, PMID: 20943583 PMC3150158

[28] World Health Organization. Mental health of adolescents. (2021). Available at: https://www.who.int/news-room/fact-sheets/detail/adolescent-mental-health. (accessed November 17, 2021).

[29] Kebede MA Anbessie B Ayano G. Prevalence and predictors of depression and anxiety among medical students in Addis Ababa. Ethiopia *Int J Ment Health Syst*. (2019) 13:30. doi: 10.1186/s13033-019-0287-6, PMID: 31080499 PMC6501290

[30] Xiong J Zhou Z Chen W You Z Zhai Z. Development of the mobile phone addiction tendency scale for college students. Chin Ment Health J. (2012) 26:222–5.

[31] Li G Xie J An L Hou G Jian H Wang W. A generalizability analysis of the mobile phone addiction tendency scale for chinese college students. Front Psych. (2019) 10:241. doi: 10.3389/fpsyt.2019.00241, PMID: 31105601 PMC6499152

[32] Kang W. Understanding the associations between the number of close friends and life satisfaction: considering age differences. Front Psychol. (2023) 14:1105771. doi: 10.3389/fpsyg.2023.1105771, PMID: 37057175 PMC10086127

[33] Thompson A Smith MA McNeill A Pollet TV. Friendships, loneliness and psychological wellbeing in older adults: a limit to the benefit of the number of friends. Ageing Soc. (2022):1–26. doi: 10.1017/S0144686X22000666

[34] American Psychiatric Association Division of Research. Highlights of changes from DSM-IV to DSM-5: Somatic symptom and related disorders focus. (2013).

[35] Kroenke K Spitzer RL Williams JB. The PHQ-9: validity of a brief depression severity measure. J Gen Intern Med. (2001) 16:606–13. doi: 10.1046/j.1525-1497.2001.016009606.x, PMID: 11556941 PMC1495268

[36] Liu Z Liu R Zhang Y Zhang R Liang L Wang Y. Latent class analysis of depression and anxiety among medical students during COVID-19 epidemic. BMC Psychiatry. (2021) 21:498. doi: 10.1186/s12888-021-03459-w, PMID: 34641795 PMC8506472

[37] Spitzer RL Kroenke K Williams JB Löwe B. A brief measure for assessing generalized anxiety disorder: the GAD-7. Arch Intern Med. (2006) 166:1092–7. doi: 10.1001/archinte.166.10.1092 16717171

[38] Wasil AR Malhotra T Nandakumar N Glinski S Bhatia A DeRubeis RJ. Which symptoms of depression and anxiety matter Most? An investigation of subjective importance ratings with college students in India. Behav Ther. (2022) 53:958–66. doi: 10.1016/j.beth.2022.04.001, PMID: 35987551

[39] Wang Q Sun W Wu H. Associations between academic burnout, resilience and life satisfaction among medical students: a three-wave longitudinal study. BMC Med Educ. (2022) 22:248. doi: 10.1186/s12909-022-03326-6, PMID: 35382810 PMC8980514

[40] Altman DG. Interaction revisited: the difference between two estimates. BMJ. (2003) 326:219. doi: 10.1136/bmj.326.7382.219, PMID: 12543843 PMC1125071

[41] Liu X Ping S Gao W. Changes in undergraduate students' psychological well-being as they experience university life. Int J Environ Res Public Health. (2019) 16:2864. doi: 10.3390/ijerph16162864, PMID: 31405114 PMC6719208

[42] Dessauvagie AS Dang HM Nguyen TAT Groen G. Mental health of university students in southeastern Asia: a systematic review. Asia Pac J Public Health. (2022) 34:172–81. doi: 10.1177/10105395211055545, PMID: 34798781 PMC8978462

[43] Lugata S Elinisa M Doshi B Kashuta RA Hango S Mallosa WJ. Symptoms and predictors of depression among university students in the Kilimanjaro region of Tanzania: a cross-sectional study. J Ment Health. (2021) 30:255–62. doi: 10.1080/09638237.2020.1793129, PMID: 32697163

[44] Tang Z Feng S Lin J. Depression and its correlation with social support and health-promoting lifestyles among Chineseuniversity students: a cross-sectional study. BMJ Open. (2021) 11:e044236. doi: 10.1136/bmjopen-2020-044236, PMID: 34226212 PMC8258557

[45] Lovell GP Nash K Sharman R Lane BR. A cross-sectional investigation of depressive, anxiety, and stress symptoms and health-behavior participation in Australian university students. Nurs Health Sci. (2015) 17:134–42. doi: 10.1111/nhs.12147, PMID: 24799077

[46] Shao R He P Ling B Tan L Xu L Hou Y. Prevalence of depression and anxiety and correlations between depression, anxiety, family functioning, social support and coping styles among Chinese medical students. BMC Psychol. (2020) 8:38. doi: 10.1186/s40359-020-00402-8, PMID: 32321593 PMC7178943

[47] Quek TT Tam WW Tran BX Zhang M Zhang Z Ho CS. The global prevalence of anxiety among medical students: a meta-analysis. Int JEnviron Res Public Health. (2019) 16:2735. doi: 10.3390/ijerph16152735, PMID: 31370266 PMC6696211

[48] Lupo MK Strous RD. Religiosity, anxiety and depression among Israeli medical students. Isr Med Assoc J. (2011) 13:613–8 PMID: 22097231 PMID: 22097231

[49] Ramón-Arbués E Gea-Caballero V Granada-López JM Juárez-Vela R Pellicer-García B Antón-Solanas I. The prevalence of depression, anxiety and stress and their associated factors in college students. Int J Environ Res Public Health. (2020) 17:7001. doi: 10.3390/ijerph17197001, PMID: 32987932 PMC7579351

[50] Sakai M Nakanishi M Yu Z Takagi G Toshi K Wakashima K. Depression and anxiety among nursing students during the COVID-19 pandemic in Tohoku region, Japan: a cross-sectional survey. Jpn J Nurs Sci. (2022) 19:e12483. doi: 10.1111/jjns.12483, PMID: 35384284 PMC9115080

[51] Zhou SJ Wang LL Qi M Yang XJ Gao L Zhang SY. Depression, anxiety, and suicidal ideation in Chinese university students during the COVID-19 pandemic. Front Psychol. (2021) 12:669833. doi: 10.3389/fpsyg.2021.669833, PMID: 34421725 PMC8375404

[52] Liu CH Stevens C Wong S Yasui M Chen JA. The prevalence and predictors of mental health diagnoses and suicide among U.S. college students:implications for addressing disparities in service use. Depress Anxiety. (2019) 36:8–17. doi: 10.1002/da.22830, PMID: 30188598 PMC6628691

[53] Barbosa-Camacho FJ Romero-Limón OM Ibarrola-Peña JC Almanza-Mena YL Pintor-Belmontes KJ Sánchez-López VA. Depression, anxiety, and academic performance in COVID-19: a cross-sectional study. BMC Psychiatry. (2022) 22:443. doi: 10.1186/s12888-022-04062-3, PMID: 35773635 PMC9243721

[54] Xiang MQ Tan XM Sun J Yang HY Zhao XP Liu L. Relationship of physical activity with anxiety and depression symptoms in chinese college students during the COVID-19 outbreak. Front Psychol. (2020) 11:582436. doi: 10.3389/fpsyg.2020.582436, PMID: 33329238 PMC7714784

[55] Brenneisen Mayer F Souza Santos I Silveira PS Itaqui Lopes MH de Souza AR Campos EP. Factors associated to depression and anxiety in medical students: a multicenter study. BMC Med Educ. (2016) 16:282. doi: 10.1186/s12909-016-0791-1, PMID: 27784316 PMC5080800

[56] Iorga M Dondas C Zugun-Eloae C. Depressed as freshmen, stressed as seniors: the relationship between depression, perceived stress and academic results among medical students. Behav Sci (Basel). (2018) 8:70. doi: 10.3390/bs8080070, PMID: 30081444 PMC6115777

[57] Abuelezam NN Lipson SK Abelson S Awad GH Eisenberg D Galea S. Depression and anxiety symptoms among Arab/middle eastern American college students: modifying roles of religiosity and discrimination. PLoS One. (2022) 17:e0276907. doi: 10.1371/journal.pone.0276907, PMID: 36327288 PMC9632767

[58] Peng P Chen S Hao Y He L Wang Q Zhou Y. Network of burnout, depression, anxiety, and dropout intention in medical undergraduates. Int J Soc Psychiatry. (2023) 69:1520–31. doi: 10.1177/00207640231166629, PMID: 37092762

[59] Bozoglan B Demirer V Sahin I. Problematic internet use: functions of use, cognitive absorption, and depression. Comput Hum Behav. (2014) 37:117–23. doi: 10.1016/j.chb.2014.04.042

[60] Odaci H Celik CB. Who are problematic internet users? An investigation of the correlations between problematic internet use and shyness, loneliness, narcissism, aggression and self-perception. Comput Hum Behav. (2013) 29:2382–7. doi: 10.1016/j.chb.2013.05.026

[61] Bandura A. Social foundations of thought and action: A social cognitive theory prentice Hall, Englewood cliffs (1985). 169 p.

[62] Zhang X Gao F Kang Z Zhou H Zhang J Li J. Perceived academic stress and depression: the mediation role of mobile phone addiction and sleep quality. Front Public Health. (2022) 10:760387. doi: 10.3389/fpubh.2022.760387, PMID: 35145942 PMC8821519

[63] Elhai JD Yang H Fang J Bai X Hall BJ. Depression and anxiety symptoms are related to problematic smartphone use severity in chinese young adults: fear of missing out as a mediator. Addict Behav. (2020) 101:105962. doi: 10.1016/j.addbeh.2019.04.020, PMID: 31030950

[64] Li Y Li G Liu L Wu H. Correlations between mobile phone addiction and anxiety, depression, impulsivity, and poor sleep quality among college students: a systematic review and meta-analysis. J Behav Addict. (2020) 9:551–71. doi: 10.1556/2006.2020.00057, PMID: 32903205 PMC8943681

[65] Zou L Wu X Tao S Xu H Xie Y Yang Y. Mediating effect of sleep quality on the relationship between problematic mobile phone use and depressive symptoms in college students. Front Psych. (2019) 10:822. doi: 10.3389/fpsyt.2019.00822, PMID: 31798473 PMC6865206

[66] Sullivan HS. The interpersonal theory of psychiatry. Fortschr Neurol Psychiatr Grenzgeb. (1958) 26:430–40. 13574290

[67] Wang M Tao FB Wu XY. Research progress on the comorbidity of anxiety and depression in children and adolescents. Chi J Prev Med. (2022) 56:1011–6. doi: 10.3760/cma.j.cn112150-20220325-00283, PMID: 35899357

[68] Milam AJ Oboh O Brown Z Edwards-Johnson J Terry A Barajas CB. Symptoms of depression and anxiety among black medical students: the role of peer connectedness and perceived discrimination. J Racial Ethn Health Disparities. (2022) 9:2180–7. doi: 10.1007/s40615-021-01157-7, PMID: 34599490 PMC8486160

[69] Horowitz LM Rosenberg SE Baer BA Ureño G Villaseñor VS. Inventory of interpersonal problems: psychometric properties and clinical applications. J Consult Clin Psychol. (1988) 56:885–92. doi: 10.1037//0022-006x.56.6.885, PMID: 3204198

[70] Kraut R Patterson M Lundmark V Kiesler S Mukopadhyay T Scherlis W. A social technology that reduces social involvement and psychological well-being? Am Psychol. (1998) 53:1017–31. doi: 10.1037//0003-066x.53.9.1017, PMID: 9841579

[71] Li C Zheng Y Tang WJ Yang FY Xie XD He JC. Mobile phone addiction levels and negative emotions among Chinese young adults: the mediating role of interpersonal problems. Comput Hum Behav. (2016) 55:856–66. doi: 10.1016/j.chb.2015.10.030

[72] Kim GS Lee CY Kim IS Lee TH Cho E Lee H. Dyadic effects of individual and friend on physical activity in college students. Public Health Nurs. (2015) 32:430–9. doi: 10.1111/phn.12176, PMID: 25565084

[73] Tao S Wu X Yang Y Tao F. The moderating effect of physical activity in the relation between problematic mobile phone use and depression among university students. J Affect Disord. (2020) 273:167–72. doi: 10.1016/j.jad.2020.04.012, PMID: 32421598

