# Supplementary material for: The impact of problematic mobile phone use and the number of close friends on depression and anxiety symptoms among college students
Source: Front Psychiatry. 2024 Jan 8;14:1281847. doi: 10.3389/fpsyt.2023.1281847 (PMC10800545; doi:10.3389/fpsyt.2023.1281847)
Supplement: Supplementary file 1 [file Table_1.DOCX]

Supplementary Material

**Table S1. The effects of PMPU and NCFs on depression and anxiety symptoms, unadjusted model.**

| Variables | Depression symptoms | |  | Anxiety symptoms | |  | Depression and anxiety symptoms | |
| --- | --- | --- | --- | --- | --- | --- | --- | --- |
|  | *OR*(95*CI*%) | *P* |  | *OR*(95*CI*%) | *P* |  | *OR*(95*CI*%) | *P* |
| PMPU |  |  |  |  |  |  |  |  |
| no | 1.00 |  |  | 1.00 |  |  | 1.00 |  |
| yes | 6.14(5.44-6.93) | <0.001 |  | 5.27(4.73-5.88) | <0.001 |  | 9.03(7.90-10.33) | <0.001 |
| NCFs |  |  |  |  |  |  |  |  |
| ≥3 | 1.00 |  |  | 1.00 |  |  | 1.00 |  |
| 1-2 | 1.52(1.38-1.67) | <0.001 |  | 1.56(1.41-1.72) | <0.001 |  | 1.69(1.52-1.88) | <0.001 |
| 0 | 1.97(1.52-2.56) | <0.001 |  | 1.96(1.52-2.53) | <0.001 |  | 2.28(1.72-3.03) | <0.001 |
| PMPU×NCFs |  |  |  |  |  |  |  |  |
| no×≥3 | 1.00 |  |  | 1.00 |  |  | 1.00 |  |
| yes×0 | 10.16(5.08-20.32) | <0.001 |  | 6.52(4.00-10.64) | <0.001 |  | 6.20(3.87-9.93) | <0.001 |
| yes×1-2 | 6.38(5.28-7.72) | <0.001 |  | 4.98(4.27-5.81) | <0.001 |  | 5.11(4.39-5.95) | <0.001 |
| Note: PMPU, problematic mobile phone use; NCFs, the number of close friends. | | | | | | | | |

**Table S2. Odds ratio of depression symptoms by level of NCFs and PMPU in females and males, and the gender ratio.**

| Variables | Females | |  | Males | |  | Ratio of two odds ratios in girls versus boys | |
| --- | --- | --- | --- | --- | --- | --- | --- | --- |
|  | *OR*(95%*CI*)* | *P* |  | *OR*(95%*CI*)* | *P* |  | *ROR** | *P* |
| PMPU |  |  |  |  |  |  |  |  |
| no | 1.00 |  |  | 1.00 |  |  | 1.00 |  |
| yes | 5.75(4.98-6.64) | <0.001 |  | 7.09(5.63-8.93) | <0.001 |  | 0.81(0.62-1.06) | 0.131 |
| NCFs |  |  |  |  |  |  |  |  |
| 0 | 2.07(1.43-2.99) | <0.001 |  | 1.78(1.20-2.65) | 0.004 |  | 1.16(0.68-2.00) | 0.585 |
| 1-2 | 1.40(1.25-1.56) | <0.001 |  | 1.65(1.38-1.97) | <0.001 |  | 0.85(0.69-1.05) | 0.124 |
| ≥3 | 1.00 |  |  | 1.00 |  |  | 1.00 |  |
| PMPU×NCFs |  |  |  |  |  |  |  |  |
| no×≥3 | 1.00 |  |  | 1.00 |  |  | 1.00 |  |
| yes×0 | 8.93(3.53-22.59) | <0.001 |  | 10.12(3.53-29.02) | <0.001 |  | 0.88(0.22-3.59) | 0.861 |
| yes×1-2 | 6.01(4.80-7.53) | <0.001 |  | 6.83(4.77-9.78) | <0.001 |  | 0.88(0.58-1.34) | 0.554 |
| Note: *Adjusting for sex, residence, only child, family economic status, parents’ education level; PMPU, problematic mobile phone use; NCFs, the number of close friends; ^*^ Calculated by adjusted odds ratio. | | | | | | | | |

**Table S3. Odds ratio of anxiety symptoms by level of NCFs and PMPU in females and males, and the gender ratio.**

| Variables | Females | |  | Males | |  | Ratio of two odds ratios in girls versus boys | |
| --- | --- | --- | --- | --- | --- | --- | --- | --- |
|  | *OR*(95%*CI*)* | *P* |  | *OR*(95%*CI*)* | *P* |  | *ROR** | *P* |
| PMPU |  |  |  |  |  |  |  |  |
| no | 1.00 |  |  | 1.00 |  |  | 1.00 |  |
| yes | 5.05(4.44-5.75) | <0.001 |  | 5.71(4.63-7.05) | <0.001 |  | 0.88(0.69-1.13) | 0.329 |
| NCFs |  |  |  |  |  |  |  |  |
| 0 | 2.03(1.43-2.87) | <0.001 |  | 1.75(1.16-2.62) | 0.007 |  | 1.16(0.68-1.98) | 0.587 |
| 1-2 | 1.48(1.31-1.66) | <0.001 |  | 1.62(1.34-1.97) | <0.001 |  | 0.91(0.73-1.15) | 0.433 |
| ≥3 | 1.00 |  |  | 1.00 |  |  | 1.00 |  |
| PMPU×NCFs |  |  |  |  |  |  |  |  |
| no×≥3 | 1.00 |  |  | 1.00 |  |  | 1.00 |  |
| yes×0 | 5.56(2.94-10.53) | <0.001 |  | 6.92(3.18-15.08) | <0.001 |  | 0.80(0.29-2.20) | 0.670 |
| yes×1-2 | 4.99(4.15-5.98) | <0.001 |  | 4.65(3.46-6.25) | <0.001 |  | 1.07(0.76-1.52) | 0.691 |
| Note: *Adjusting for sex, residence, only child, family economic status, parents’ education level; PMPU, problematic mobile phone use; NCFs, the number of close friends; ^*^ Calculated by adjusted odds ratio. | | | | | | | | |

**Table S4. Odds ratio of the comorbidities of depression and anxiety symptoms by level of NCFs and PMPU in females and males, and the gender ratio.**

| Variables | Females | |  | Males | |  | Ratio of two odds ratios in girls versus boys | |
| --- | --- | --- | --- | --- | --- | --- | --- | --- |
|  | *OR*(95%*CI*)* | *P* |  | *OR*(95%*CI*)* | *P* |  | *ROR** | *P* |
| PMPU |  |  |  |  |  |  |  |  |
| no | 1.00 |  |  | 1.00 |  |  | 1.00 |  |
| yes | 5.36(4.71-6.10) | <0.001 |  | 5.83(4.72-7.21) | <0.001 |  | 0.92(0.72-1.18) | 0.507 |
| NCFs |  |  |  |  |  |  |  |  |
| 0 | 2.09(1.48-2.96) | <0.001 |  | 1.87(1.24-2.81) | 0.007 |  | 1.12(0.65-1.91) | 0.684 |
| 1-2 | 1.50(1.33-1.69) | <0.001 |  | 1.54(1.26-1.87) | <0.001 |  | 0.97(0.77-1.23) | 0.823 |
| ≥3 | 1.00 |  |  | 1.00 |  |  | 1.00 |  |
| PMPU×NCFs |  |  |  |  |  |  |  |  |
| no×≥3 | 1.00 |  |  | 1.00 |  |  | 1.00 |  |
| yes×0 | 4.66(2.56-8.50) | <0.001 |  | 7.88(3.62-17.19) | <0.001 |  | 0.59(0.22-1.58) | 0.295 |
| yes×1-2 | 5.19(4.34-6.22) | <0.001 |  | 4.60(3.43-6.16) | <0.001 |  | 1.13(0.80-1.59) | 0.491 |
| Note: *Adjusting for sex, residence, only child, family economic status, parents’ education level; PMPU, problematic mobile phone use; NCFs, the number of close friends; ^*^ Calculated by adjusted odds ratio. | | | | | | | | |
